# Effects of mobile phone use on specific intensive care unit devices

**DOI:** 10.4103/0972-5229.45077

**Published:** 2008

**Authors:** Nidhi Hans, Farhad N. Kapadia

**Affiliations:** **From:** P.D. Hinduja Hospital and Medical Research Center, Veer Savarkar Marg, Mahim, Mumbai - 400 016, India

**Keywords:** Electromagnetic interference, mobile phone and infusion pumps, mobile phone and ventilator, mobile phones in ICU setting

## Abstract

**Background and Aims:**

To observe the effects of mobile phone use in the vicinity of medical devices used in a critical care setting.

**Subjects and Methods:**

Electromagnetic interference (EMI) was tested by using two types of mobile phones – GSM and CDMA. Mobile phones were placed at a distance of one foot from three medical devices – syringe pump, mechanical ventilator, and the bedside monitor – in switch off, standby, and talking modes of the phone. Medical devices were observed for any interference caused by the electromagnetic radiations (EMR) from the mobile phones.

**Results:**

Out of the three medical devices that were tested, EMI occurred while using the mobile phone in the vicinity of the syringe pump, in the ‘talk mode.’ The mean variation observed in the calculated and delivered volume of the syringe pump was 2.66 ml. Mechanical ventilator did not show any specific adverse effects with mobile phone use in the one-foot vicinity. No other adverse effects or unexplained malfunctions or shutdown of the syringe pump, mechanical ventilator, or the bedside monitor was noted during the study period of 36 hours.

**Conclusion:**

EMI from mobile phones have an adverse effect on the medical devices used in critical care setup. They should be used at least one foot away from the diameter of the syringe pump.

Cellular phone usage has grown exponentially in the recent years with introduction of new communication systems and newer and smaller phone models. Intensive Care Units (ICU) in hospitals are also increasingly using sophisticated electronic medical devices. Mobile phones are often used by ICU personnel and occasionally by relatives and patients in the near vicinity of electronic medical equipment. The aim of this study was to evaluate the Electromagnetic Interference (EMI) of currently popular cellular phones, which work based on either Global System of Mobile Communication (GSM) or Code Division Multiple Access (CDMA) technologies, in the immediate vicinity of electronic medical devices used in the ICU settings.

## Materials and Methods

The study was conducted in two phases at the P.D. Hinduja National Hospital and Medical Research Centre, Mumbai. In the first Side room phase, the medical devices tested were intravenous infusion syringe pumps (B BRAUN) and mechanical ventilators (VERSA MED). In the second Bedside phase, a four-channel bedside monitor (Philips-Intellivue MP40) was studied. The traces displayed were an ECG trace with calculated pulse and respiratory rates, pulse oximetry, and two invasive pressure traces, from a central venous catheter and arterial catheter. The GSM-based mobile phonesused were Motorola V3i and Nokia 6600 and Nokia 5310 with Nokia HS-4W bluetooth device. The CDMA phone used was a LG 5130.

These mobile phones were tested in three different modes:

1. Switched off mode (tested for 30-minutes duration).
2. Switch on but not in use, that is, standby mode (tested for 30-minute duration).
3. Talk mode (tested for 10-minute duration).

GSM mobile phones were also tested in the talk mode linked to the bluetooth device in addition to the above mentioned modes.

The medical devices were first verified to be operating normally. The GSM mobile phone in switched off mode was placed at a distance of one foot in level with the medical device and started as detailed below. Recordings were made every 10 minutes for a total of 30-minutes duration. After each recording, at the end of 30 minutes, the medical device was restarted. The study was then repeated with CDMA mobile phone and the other phones in different modes, except in the talk mode, where the call durations of varying lengths were recorded in the 10-minute period. All studies were re-recorded two additional times. Mobile phones in the switched off mode was taken as control.

### Syringe Pump

To test mobile phones in ‘switched off’ and ‘switched on’ modes, the syringe pump was set at two different rates of 40 ml per hour (low flow rate) and 200 ml per hour (high flow rate) with a 50 ml syringe filled with 5% dextrose, and each recording noted. To test mobile phones in the talk mode, syringe pump was set at 200 ml per hour. It was specifically observed for changes in the infusion rate, i.e. whether occurring at a faster or slower rate, and the plunger moving in the opposite direction.

### Ventilator

Ventilator was set at respiratory rate of 16 breaths per minute, and a tidal volume of 500 ml. FiO_2_ was monitored with the ventilator internal analyzer and an external FiO_2_ analyzer calibrated to 21% oxygen and attached to the breathing circuit. Mechanical ventilator was specifically observed for changes in the respiratory rate (RR), tidal volume (TV), or measured FiO_2_.

### Bedside monitor

The mobile device was placed on a stand at a distance of one foot from the front display of the monitor or was placed on top of the monitor. One study was done on a stable patient recovering from malaria, hyperpyrexia, and shock. The patient only had ECG and pulse oximetry monitoring during the study. The other study was on a patient with gastrointestinal sepsis with shock and multiorgan failure. The patient was on mechanical ventilation, inotropes and vasopressors, intermittent hemodialysis, and other standard therapies for severe sepsis as per the surviving sepsis guidelines. Informed consent was obtained from the patient or the family, for the in-cubicle study of the bedside monitor. The study was approved by the Scientific and Ethics Committee of the hospital.

All the medical devices tested were also observed for, switching off of the device, any alteration in the set parameters or any other unexplained variation. All timings were accurately measured and maintained using a stopwatch.

## Results

The total study time was 36 hours (control mode for 15 hours, standby mode for 15 hours, and talk mode for 6 hours).

### Syringe pump

Syringe pump was studied for a total duration of 13.5 hours (with control mode for 6 hours, standby mode for 6 hours, and talk mode for 1.5 hours). The recordings of syringe pump volumes delivered. The differences between set and delivered parameters were similar when control to standby and talk modes with syringe pump set at 40 ml/hour was compared. With the syringe pump set at 200 ml/hour, during the control mode the mean variation was 0.45 ml and the maximum variation was 1.54 ml, whereas in the standby mode the mean variation was 0.2 ml and the maximum variation was 0.7 ml. During the talk mode, for call durations ranging from 1 minute and 40 seconds to 14 minutes and 30 seconds, we noted a mean variation in calculated and delivered volume of 2.66 ml, with the range of 0.7–6.5 ml. We did not note any other adverse effects, unexplained malfunctions, or shutdown of the syringe pumps.

### Mechanical ventilator

Mechanical ventilator was studied for a total duration of 7.5 hours (with control mode for 3 hours, standby mode for 3 hours, and talk mode of 1.5 hours). In ventilator, there was no effect seen on the set RR or FiO_2_ in all modes of phones. Variation was seen in the TV in all the phone modes. During the control mode, mean variation was 38.33 ml and the maximum was 56 ml. Mean variation seen during the standby mode was 32.39 ml and maximum variation was 38 ml, whereas during the talk mode the mean variation was 44.22 ml and the maximum variation was 78 ml. We did not note any other adverse effects, or unexplained malfunctions, or shutdown of the mechanical ventilator.

### Bedside monitor

This phase was studied for a total duration of 15 hours (with control mode being for a duration of 6 hours, standby mode for 6 hours, and talking mode for 3 hours). We did not note any other adverse effects or unexplained malfunctions or shutdown of the bedside monitor. Additionally, in the second patient, no variations or malfunctions were noted with the syringe pumps, mechanical ventilator, or hemodialysis machine.

## Discussion

A Mobile phone is a long-range, portable electronic device used for mobile communication that utilizes a network of specialized base stations known as cell sites. The current mobile phone also provides the user with additional features such as SMS for text messaging, MMS for sending and receiving photos and videos, E-mails and packet switching for access to the Internet. Most current mobile phones connect to a cellular network of base stations, which in turn are interconnected to the public switched telephone network (the exception being satellite phones). First-generation phones were mainly used for voice transmission, while the second- and third-generation mobile phones enable us to use wireless Internet access (GPRS, MMS, bluetooth, infrared services, etc). Mobile phones in current use are of GSM and CDMA types. The GSM requires a SIM card and uses frequency range of 900–800 MHz. The CDMA uses many simultaneous transmitters on the same frequency. (http://en.wikipedia.org/wiki/GSM and http://en.wikipedia.org/wiki/CDMA2000).

There are certain issues concerning the EMR from mobile phones. Firstly, the amount of EMR[1] emitted, and secondly the distance from which they should be used to prevent any effect on the medical device. Wireless phones emit low levels of EMR in the microwave range while being used.[2] They also emit very low levels of EMR during the standby mode.

Also, an issue of concern is the construction of electronic devices and its capacity to withstand the EMR without producing any adverse effects. The current standards, set by the United States Food and Drug Administration in 1979, has specified that medical equipment should be immune from interference in fields of up to 7 V/m within the frequency range of 450–1000 MHz. Most modern electronic equipments are shielded from radiofrequency waves. However, certain devices may not be shielded against the radiofrequency waves, catalogue that came along with the CDMA mobile phone model number 5130. This could be due to very rapid transition in the production of mobile phones and use of newer and newer technologies to incorporate maximum facilities in the mobile phones.

Effects of mobile phone interference with the medical devices have been currently graded as clinically relevant EMI; hazardous incidents and incidents requiring intervention are graded on an adjusted scale of critical care adverse effects prepared by two board-certified and experienced intensivists.[3] Recently Remko *et al* have assessed and classified incidents of EMI on critical care equipment in their study.[4]

None of these have a clear definition which is standardised to reach to a conclusion for usage of mobile phones in the vicinity of medical devices. Inspite of having no clear definitions, mobile phones are ranked second, after two-way radios used by emergency crews, in causing the highest risk of interference.[5]

The current safe distance for the use of mobile phones is said to be one meter, as proposed by Irnich and Tobisch[6] and ECRI.[7] This is of particular concern, especially in critical areas such as ICUs, operating theatres, special-care nurseries, etc.

Problems with EMI of medical devices have been known for some time in hospitals. Research group, manufacturers, and governmental and nongovernmental agencies have reported incidents related to EMI.[8] These studies have reported problems attributed to EMI from mobile phones with medical devices such as syringe pumps, ventilators, external defibrillators, monitors, etc. Prompted by these reports, certain recommendations were put up to restrict the use of mobile phones in critical areas of the hospital. These recommendations include either the definition of separation distance or complete banning of mobile phones. In view of lack of evidence, restrictions have been criticized.[9]

There are two incidents reported, one leading to the patient's death due to the respirator being shut off and the other being acute epinephrine poisoning with mobile phone use in the vicinity of the medical device. Acute epinephrine poisoning was reported in an 18-year-old man treated with epinephrine infusion (22 mg in 50 ml D5W) admitted for septic shock with unstable vitals. Few hours after titration of the drug, the patient complained of signs and symptoms of catecholamine excess. Later, the hospital investigating team found out that it was due to use of mobile phone in the vicinity of syringe pump triggering its malfunction. This was confirmed by using mobile phones at varying distances from syringe pump set at 30 ml per hour. During several test calls, the syringe pump spontaneously delivered at the rate of 999 ml per hour (maximum volume delivered by the syringe pump). The effect of this will depend on the drug that is being infused and the condition of the patient. This malfunction was not recorded in the history log of infusion pump.[10] These are very rare events but lethal ones, which have a wide range of effects, varying from just a nuisance to life-threatening consequences. These may take just a minute or few seconds to make their effects evident.

In conclusion, EMI from mobile phones have an adverse effect on the medical devices used in critical care setup. They should be used at least one foot away from the diameter of the syringe pump.
